# A Clinical Lecture on Acute Pulmonary Phthisis

**Published:** 1852-05

**Authors:** David W. Yandell

**Affiliations:** One of the Consulting Physicians to the Louisville Marine Hospital; Louisville, Ky.


Art. IV.—*A Clinical Lecture on Acute Pulmonary Phthisis.* By David W. Yandell, M. D., one of the Consulting Physicians to the Louisville Marine Hospital.

At our last meeting I concluded with the remark, that the blister which was applied to Ben's chest had given rise to so much suffering, that I did not think it advisable to enjoin its repetition. The chest is not considered the most appropriate seat for counter-irritation in phthisis; and it was through the mistake of our patient's wife, that the flies were applied in this situation. I directed her to put them on his arm, and was not a little annoyed that she had neglected to do so, as it obliged me to suspend all physical exploration until the vesicated surface had healed. This required four days. During this interval the mucous rhonchus had given place, at the summit of the lung, to the cavernulous rhonchus, and had shifted its locality to that portion of the lung which, a week before, presented the signs characteristic of the first stage of phthisis. How rapidly had these changes been effected! With what alarming celerity had tubercles passed through their several stages of development, from the crude and rudimentary deposit, to softening and the formation of anfractuosities. Day by day Ben wasted away. At almost every examination, the mucous and cavernulous rhonchi were found to have advanced a little downward, and fresh portions of lung to be invaded by the disease.

Such, gentlemen, is by no means the ordinary course of even acute phthisis. Usually, a crop of tubercles is formed which slowly or rapidly pass through their stated periods—soften, ulcerate, and destroy. Here, for the time being, the work ends—the process stops. Sooner or later the morbid action is renewed: a fresh crop of tubercles is deposited, and runs the course of the first. The process of deposition is not continually going on. A portion of lung, almost entirely free from tubercle, will often be found in the immediate neighborhood of large masses of this formation. In this case, however, the work of deposition kept pace with that of softening, and almost every day we could detect an increase in the amount of both. This is a very uncommon and remarkable circumstance. Some of you have seen, in the hospital and in my private practice, cases of chronic phthisis, in which the disease seemed really to intermit—cases in which you were able to tell, almost to the day, when a portion of lung, hitherto healthy, had become infiltrated with tubercle; and after this had been accomplished, you have seen these cases improve, grow decidedly better, and the patients fancy themselves almost well. Now, in such instances it is impossible to doubt that, for some reason or other, the process of tuberculization has, for the time being, been put an end to. No such good fortune favored our patient. The progress of the disease with him was incessantly onward ; and without dwelling longer upon this portion of our subject, I will simply state, that he died within forty days of the time that he presented the first physical signs of phthisis.

There are many cases on record where phthisis has terminated in a shorter time than this. In 1846, I communicated to this journal, notes of a case in Louis' wards, in the Hotel Dieu, at Paris, that ended fatally in about twenty-days. The autopsy revealed numerous granulations of the left lung, especially at its summit, and a considerable crop of tubercles, in the crude state, of very small volume. The right lung contained a large cavern in the summit, which M. Louis thought of recent formation. Numerous granulations and tubercles, some in the crude state, others beginning to soften, were observed throughout the entire extent of the organ. This was the first example of acute phthisis that I ever saw. Ben's case was the second. M. Louis has stated, in his work on pulmonary phthisis, the case of a man who died in twenty days after the first signs of tubercles were observed; and yet, in this short space of time, excavations had been formed in the lung. Andral and Portal mention cases which have run their course in eleven days, and numerous instances are reported of individuals who have died of phthisis within three weeks.

The diagnosis of acute phthisis is seldom an easy, often a very difficult, and sometimes an impracticable task. In that variety where gray granulations accumulate generally through the lung, giving rise to asphyxia, and usually proving fatal before suppuration has taken place, the diagnosis is extremely difficult. It may be distinguished from bronchitis by the absence of the characteristic rhonchi at the inferior parts of the lung, a larger amount of dyspnea, and a want of the full muco-purulent expectoration that is so invariably present in the second period of this disease. There is but little likelihood of its being confounded with pleurisy. The condition of the respiration and state of the pulse, might give rise to the opinion that pneumonia was present; but "the lapse of twenty-four hours will prove that common idiopathic pneumonia does not exist, for the signs of hepatization are not an iota more obvious than the previous day." You see from this, gentlemen, that the difficulty of diagnosis is not the result of the similarity of this affection to the ordinary pulmonary inflammations, but it lies beyond this, and, according to Dr. Walshe, consists in distinguishing miliary phthisis from typhoid fever. The resemblance between these two diseases is often so great, that the shrewdest observers sometimes mistake one for the other. Dr. Walshe states that he himself committed the error of diagnosticating typhoid fever with secondary pneumonia in an individual who died a few days after with acute phthisis. "Dyspnea, prostration, bronchitic rhonchi, duskiness of face, febrile action, dry skin, adynamic state of the tongue, delirium, and stupor, exist in both affections, and may do so to similar amounts." "The abdominal symptoms and the peculiar eruption of typhoid fever draw the line, to all appearance, positively—but only in appearance: for abdominal symptoms may be present in acute phthisis, if the intestine be undergoing acute tuberculization; and although eruption probably exits in all cases of typhoid fever it certainly escapes detection, (probably from its slight amount,) in a few instances."

In acute suppurative phthisis, the diagnosis is rendered comparatively easy by the absence of the ordinary signs of pleurisy, intense bronchitis, and pneumonia, the extreme violence of the general symptoms, contrasted with the small amount of pulmonary disturbance, the general and steadily progressive loss of sonorousness of the chest, coupled with the auscultatory phenomena of destruction of tissue.

Of course, in that variety of phthisis described by Andral, in which the symptoms were remarkable as not being those usually present in this affection, I am unable to furnish you with any diagnostic guides. I am not aware that there are any. Andral states that he has seen persons, having for some time a very slight cough, who were seized with the symptoms of pneumonia or pleuritis, and that several of the individuals in whom these symptoms were observed, sank rapidly, and large masses of tuberculous matter were found in their lungs. Under such circumstances you could but diagnosticate inflammation of the substance or investing membrane of the lung, and would be destined to find at the autopsy softened tubercles instead of pneumonia or pleurisy. I am satisfied myself, however, that this error can arise only where the tubercles do not communicate with the bronchi, which must be an exceedingly rare occurrence. Wherever the ulcerative process has extended to the walls of the bronchial tubes, and the softened tuberculous mass finds its way—as it necessarily will do—into their cavities, the mucous rhonchus will inevitably be produced.

Many, perhaps most cases of acute phthisis, are to be ranked in the first variety of Dr. Walshe, in which the progress of the disease, except in regard to its rapidity, is the counterpart of that observed in chronic phthisis. It is hardly necessary to say, that in such instanees, the diagnosis is unmistakeably clear, and requires no description at my hands. During the first few days of Ben's sickness, both the rational and physical signs were of a nature to awaken doubt as to the true character of his disease. Soon, however, what had been obscure became clear; what had been doubtful became certain; what had been unlike phthisis, assumed a most striking resemblance to it,—indeed, declared itself unequivocally to be phthisis. In addition to the symptoms I have already mentioned, I should not neglect to refer to one alluded to by Dr. Stokes, which is, the resistance of the affection to treatment, even of the most active and varied description. I have alreday announced the result of our case. Ben died in the last stage of emaciation, and seemingly from the mere violence and intensity of the disease. Owing to the unwillingness of his wife, I did not open his body. The treatment that I adopted was not only that which is usually pursued in acute phthisis, but I conjoined with it that remedy so justly celebrated in the chronic form of the disease—cod-liver oil. The progress of galloping consumption is so frightfully rapid, that we can not, as so often done in chronic phthisis, follow the example of Celsus and advise a voyage to Egypt, nor that ot Aretæus who insisted upon his patients boating or sailing; nor have we the means, even if we thought it useful, to establish an artificial marine atmosphere with the fresh sea weed, as was practised by M. Laennec. Nor can we recommend a residence—which, to say the least would not be pleasant in these days of invasion—in Cuba, which you know was quite the fashion, a few years since, among American physicians; nor, on the other hand, can we insist on our patient's taking up his abode on the shores of the northern lakes, as is recommended by the author of the "Diseases of the Interior Valley." We are unable to adopt any one of these popular expedients for ridding ourselves of incurable cases. An individual suffering with acute phthisis is, usually, wholly unable to undertake such journeys, and if he wishes to die among friends and kindred, it is fortunate that he is so.

Louisville, Ky., March, 1852.

